# Kinetic and Structural Insights into β-Cyclodextrin Complexation with Asparagine Enantiomers: An Experimental and Theoretical Study

**DOI:** 10.3390/molecules30030523

**Published:** 2025-01-24

**Authors:** Constantine Kouderis, Stefanos Tsigoias, Panagiota Siafarika, Angelos G. Kalampounias

**Affiliations:** 1 Physical Chemistry Laboratory, Department of Chemistry, University of Ioannina, 45110 Ioannina, Greece; 2 Institute of Materials Science and Computing, University Research Center of Ioannina (URCI), 45110 Ioannina, Greece

**Keywords:** asparagine, enantiomers, inclusion complex, β-cyclodextrin, ultrasonic relaxation spectroscopy, IR, DFT, molecular docking

## Abstract

We report on the dynamic interactions between β-cyclodextrin (β-CD) and each one of the two enantiomers of asparagine (d-Asp, l-Asp). Molecular docking methodologies were applied to elucidate the formation of the β-CD—d-Asp and β-CD—l-Asp inclusion complexes. Ultrasonic relaxation spectra revealed a single relaxation process in the frequency range studied that is attributed to the complexation between β-CD and asparagine enantiomers. Kinetic parameters and thermodynamic properties for each system were determined directly from the concentration- and temperature-dependent acoustic measurements, respectively. Both β-CD—d-Asp and β-CD—l-Asp systems revealed subtle differences in their thermodynamic and kinetic properties. The infrared absorption spectra of the host molecule, the guest enantiomers, and both inclusion complexes were recorded to verify and further elucidate the complexation mechanism. DFT methodologies were performed to calculate the theoretical IR spectra of the inclusion complexes and compared with the corresponding experimental spectra. The close resemblance between the experimental and theoretically predicted IR spectra is supportive of the formation of inclusion complexes. The encapsulation of asparagine enantiomers in β-cyclodextrin enables not only applications in drug delivery but also the detection and separation of chimeric molecules.

## 1. Introduction

Cyclodextrins are categorized according to the number of glucopyranose units of which they are composed, and the most common cyclodextrins are α-cyclodextrin, β-cyclodextrin, and γ-cyclodextrin, which are composed of six, seven, and eight glycosidic rings, respectively. These rings are linked together by an α-(1,4)-glucosidic linkage [1]. Among these, β-cyclodextrin is the most widely used due to its cost-effectiveness and broad range of applications. Cyclodextrins are known for their ability to encapsulate small molecules within their inner cavity, a property that stems directly from their unique structure. These cyclic oligosaccharides feature an outer hydrophilic surface and a hydrophobic inner cavity, allowing them to naturally enclose small hydrophobic molecules. This inclusion process is driven by various non-covalent interactions, such as hydrogen bonds, van der Waals interactions, hydrophobic interactions, and electrostatic forces. This unique ability for molecular inclusion has enabled cyclodextrins to find widespread application across various industries, including cosmetics, the food industry, and pharmaceuticals.

Cyclodextrins, due to their ability to encapsulate specific molecules in their inner cavity, have also been used for the detection and separation of chiral molecules, such as amino acids [2,3]. Different configurations of chiral molecules exhibit differences in polarity and size. Therefore, they also present variations in encapsulation kinetics and complex stability, which allows for their separation. There can be cases where one molecule shows no encapsulation, while the other chiral counterpart forms a strong complex [4]. Amino acids are known for their chirality, as most exist in two enantiomeric forms, namely the l- and the d-form. These mirror-image isomers arise from the presence of a chiral center, typically the alpha carbon, to which different groups are attached. The l-form is the most common in nature and is primarily used in protein synthesis, while the d-form is less prevalent, although it plays specialized roles in some biological processes [5].

l-asparagine is one of the twenty proteinogenic amino acids involved in protein synthesis and is classified as a non-essential amino acid. Most mammals can synthesize it with the aid of aspartate and glutamine using the asparagine synthetase enzyme [6]. As a nitrogen-rich molecule, it plays a crucial role in nitrogen storage and transport throughout the food chain [7]. Moreover, asparagine has various uses in other biological procedures as it participates in numerous metabolic pathways. It plays a key role in the urea cycle, regulating the conversion of toxic ammonia to urea [8]. In recent decades, l-asparagine has garnered attention in cancer research as some cancer cells have a high demand for l-asparagine due to their rapid growth and proliferation. Inhibiting the metabolic path of asparagine has been explored as a potential therapeutic process [9,10]. In addition, asparagine is used as a precursor for molecules involved in neurotransmitter signaling. Studies have also shown that the concentration of l-asparagine is linked with depression in elderly people [11].

d-asparagine, the enantiomer of l-asparagine, is a non-essential amino acid. While its applications are more limited compared to its l-form, recent studies have explored the role of d-asparagine as a neuroprotective agent. Specifically, it has been found to inhibit the activity of certain enzymes involved in the production of excitatory neurotransmitters like glutamate, potentially reducing excitotoxicity, a key factor in neurological disorders and neurodegenerative diseases [6]. Additionally, d-asparagine acts as a precursor for molecules with antimicrobial properties [7] and plays a role as a starting material in the synthesis of peptides.

The formation of inclusion complexes between β-cyclodextrin and small molecules can be studied by UV–Vis, NMR, and FT infrared spectroscopy. Nevertheless, ultrasonic relaxation spectroscopy has been proven as a powerful tool in the study of inclusion complexes, providing important information concerning the kinetic and thermodynamic characteristics of the complexation mechanism [12]. In this study, the formation of inclusion complexes between β-cyclodextrin and both the d- and l-forms of asparagine was investigated using ultrasonic relaxation and vibrational spectroscopies. Additionally, molecular docking and density functional theory (DFT) were employed to provide a deeper understanding of the interactions within the system [13].

**Figure 1:**
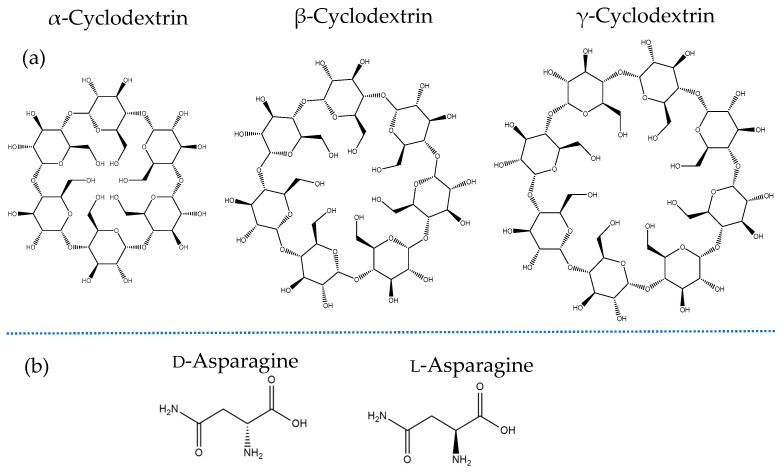
The structure of α-, β-, and γ- cyclodextrin (**a**) and the enantiomers of asparagine (**b**).

This combination of experimental and theoretical approaches provides a dynamic approach to studying the interactions between asparagine enantiomers and β-cyclodextrin. Also, it allows for a comprehensive analysis of the formation properties of the inclusion complexes reported in this study. The differences in thermodynamic and kinetic behavior of the two enantiomers during the formation of the inclusion complex may be useful for chiral recognition advancements, which is a critical step in pharmaceutical and biochemical applications [14] (Figure 1). Moreover, the encapsulation of asparagine in the β-cyclodextrins cavity shows promising applicability for drug delivery, offering controlled and targeted delivery of amino acids, which may be valuable in anti-cancer treatments.

## 2. Results and Discussion

### 2.1. Molecular Docking Between the Enantiomeric Forms of Asparagine and β-Cyclodextrin

**Figure 2:**
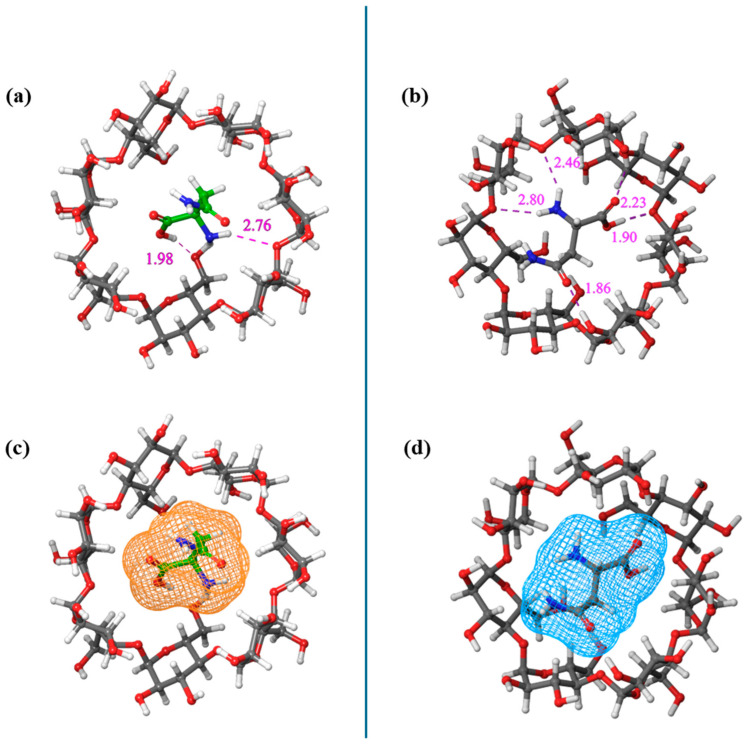
The structure of the β-CD—d-Asp (**a**) and β-CD—l-Asp (**b**) inclusion complexes after molecular docking calculations. The position and the length of the hydrogen bonds are marked in the schemes. The surface area of the guest molecules in both inclusion complexes (**c**,**d**) provides a clearer view of the guest molecules’ positioning within the cavity of β-cyclodextrin.

To examine the possibility of complex formation between each of the enantiomeric forms of asparagine and the β-cyclodextrin molecule, we performed molecular docking calculations by employing as the input the optimized structures of the corresponding molecules. The results are illustrated in Figure 2, where it seems that both asparagine enantiomers interact with β-CD through hydrogen bonds.

The free binding energies calculated for both inclusion complexes were estimated by molecular docking. For the d-asparagine complex, it was found to equal −3.86 kcal/mol, and for l-asparagine complex, it was equal to −3.81 kcal/mol, implying greater stability for the d-asparagine complex even though the l-asparagine complex showed more hydrogen bonds. A lower binding energy indicates a stronger affinity between the two molecules. The number of hydrogen bonds and the presence of steric effects strongly affect binding energy and, thus, the stability of the inclusion complex [15]. The higher the number of hydrogen bonds, the lower the binding energy is, while stronger steric effects have exactly the opposite outcome. The fact that the d-asparagine complex was found to be more stable, despite the lower total number of hydrogen bonds compared to the l-asparagine complex, is probably due to the steric effects that occur upon binding. Regarding the 1:1 stoichiometry of the resulting complex, molecular docking simulations also explored other possible stoichiometries (1:2 and 2:1). In order to examine those possibilities, a step-by-step approach was used. Firstly, molecular docking was performed between one β-cyclodextrin and one asparagine molecule to form an initial complex. This complex was then treated as the host to a second docking procedure. In this later docking, an additional β-cyclodextrin or asparagine molecule was introduced as the guest molecule to examine alternative stoichiometries. However, these configurations were found to be unfavorable, as their free binding energies were more positive compared to the 1:1 complex discussed here.

Molecular docking is a powerful computational tool in drug design and biomolecular interaction studies, but it has notable limitations. A major challenge lies in its simplified representation of molecular systems, often treating the receptor and ligand as rigid entities. This approach neglects the intrinsic flexibility of these molecules in biological contexts, potentially leading to inaccuracies when the receptor undergoes significant conformational changes upon ligand binding. Another limitation arises from docking’s reliance on scoring functions, which are based on empirical or force-field approximations. These scoring functions may fail to account for complex interactions, such as solvent effects, thereby overlooking critical interactions that occur in aqueous environments. Additionally, the sampling of ligand and receptor conformations is inherently limited; docking algorithms might not explore all possible poses or binding modes, resulting in suboptimal predictions. Furthermore, the accuracy of docking results heavily depends on the quality of the input structures. Errors in molecular structures can significantly compromise the reliability of the outcomes. These limitations underscore the need to complement docking with other computational methods for more accurate predictions. The solution to these limitations can be found by employing either molecular dynamic or DFT calculations, which can provide better results. In our study, we addressed these challenges by incorporating density functional theory (DFT) calculations. Using the B3LYP functional, we optimized the geometries of the individual molecules and their complex to ensure high-quality input and output structures. Moreover, the asparagine enantiomer was modeled with the maximum number of rotatable bonds, generating a wide range of conformations within the β-cyclodextrin cavity. This approach improved the accuracy of docking predictions and binding scores.

### 2.2. Ultrasonic Sensing of the Relaxation Process

**Figure 3:**
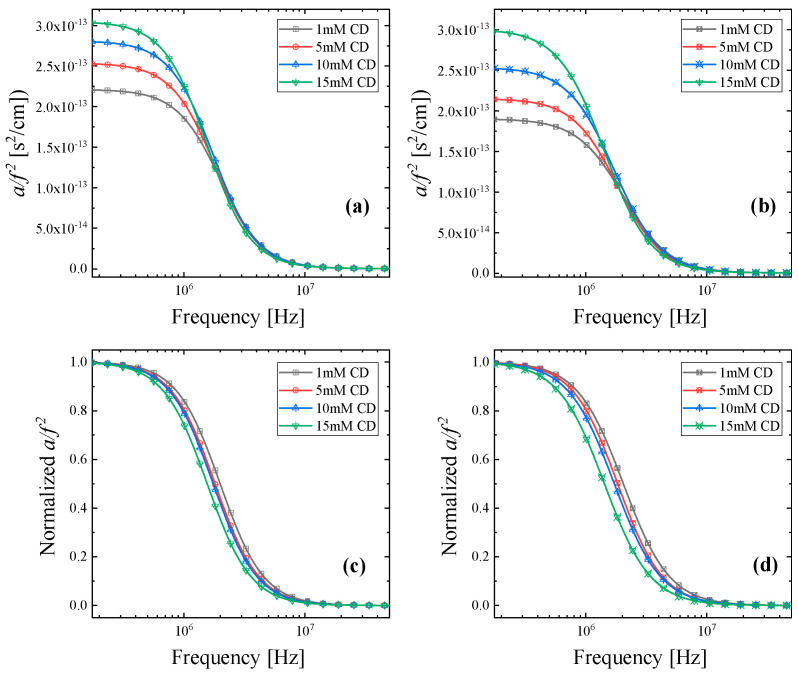
Sound absorption coefficient (*a*/*f*^2^) as a function of frequency for all concentrations studied for d-asparagine (**a**) and l-asparagine (**b**) solutions with β-cyclodextrin. Normalized *a*/*f*^2^ values as a function of frequency for d-asparagine (**c**) and l-asparagine (**d**). The normalized representation facilitates the observation of the characteristic frequency (*f_r_*) shift with β-cyclodextrin concentration in the solution.

Figure 3 shows the ultrasonic relaxation spectra measured from d-asparagine (a) and l-asparagine (b) aqueous solutions with β-cyclodextrin. The results reveal that in the low-frequency region, the sound absorption coefficient (*a*/*f*^2^) increases with β-CD concentration in the solution. The total absorption per squared frequency (α/*f*^2^) is the sum of two different contributions. The first one is referred to as the classical part and the second one as the excessive or relaxing part. The plot *a*/*f*^2^ vs. *f* is used to demonstrate these results since a straight line parallel to the frequency axis will appear when no relaxation process is observed. In contrast, if a relaxation process occurs, an excess contribution will emerge, and the overall spectrum appears as a sigmoidal function.

Usually, the experimental absorption coefficient follows a Debye-type behavior with frequency.

(1) $${\left(\frac{a}{f^{2}}\right)}_{experimental}=\sum \frac{A_{i}}{1+{\left(\frac{f}{f_{r_{i}}}\right)}^{2}}+{\left(\frac{a}{f^{2}}\right)}_{classical}=\sum \frac{A_{i}}{1+{\left(\frac{f}{f_{r_{i}}}\right)}^{2}}+B$$

The subscript *i* represents the *i-th* process that may be present in the system. Parameters *A_i_* and *f_ri_* denote the amplitude and the characteristic frequency of the corresponding relaxation. The characteristic relaxation frequency is the midpoint of the Debye function. The constant *B* represents the frequency-independent classical absorption, which includes the contribution of vibrational relaxation, visco-thermal absorption, and radiation [16].

To facilitate the observation of the frequency shift with concentration, we present in Figure 3c,d the normalized *a*/*f*^2^ values. Indeed, a clear red shift is observed with β-CD concentration, and only a single relaxation process (*i* = 1) is observed for both systems, which is attributed to the inclusion complex formation mechanism. The continuous lines in Figure 3 represent the total relaxation curves, while symbols denote the experimental data. The goodness of fit indicates that the Debye distribution function sufficiently describes the experimental data.

In both cases, the addition of β-cyclodextrin leads to an increase in relaxation amplitude along with a corresponding shift in relaxation frequency toward lower values. This behavior is characteristic of the complex formation mechanism. As the concentration of β-cyclodextrin increases, the inclusion effect becomes more pronounced [17].

The complexation mechanism between β-cyclodextrin and amino acid systems has a 1:1 stoichiometry [18]. The 1:1 stoichiometry was also confirmed theoretically by our molecular docking calculations, as already discussed. Thus, in the case of asparagine enantiomers, the equations representing these reactions are:

(2) $$\beta -CD+D-Asp\begin{array}{c} k_{f,D}\\ ↔\\ k_{b,D} \end{array}\beta -CD—D-Asp$$

and

(3) $$\beta -CD+L-Asp\begin{array}{c} k_{f,L}\\ ↔\\ k_{b,L} \end{array}\beta -CD—L-Asp$$

Parameters *k_f,D_*, *k_b,D_* and *k_f,L_*, *k_b,L_* correspond to the forward and backward rate constants of the reaction for the d- and l-enantiomers, respectively.

The respective kinetic equations are [19]:

(4) $$2\pi f_{r,D}=k_{f,D}\left({\left[\beta -CD\right]}_{eq}+{\left[D-Asp\right]}_{eq}\right)+k_{b,D}\;$$

and

(5) $$2\pi f_{r,L}=k_{f,L}\left({\left[\beta -CD\right]}_{eq}+{\left[L-Asp\right]}_{eq}\right)+k_{b,L}$$

where [β-CD]_eq_, [d-Asp]_eq_, and [l-Asp]_eq_ represent the concentration of β-cyclodextrin, d-asparagine, and l-asparagine at equilibrium, respectively. The activities of the molecules are considered equal to unity due to their low concentration.

**Figure 4:**
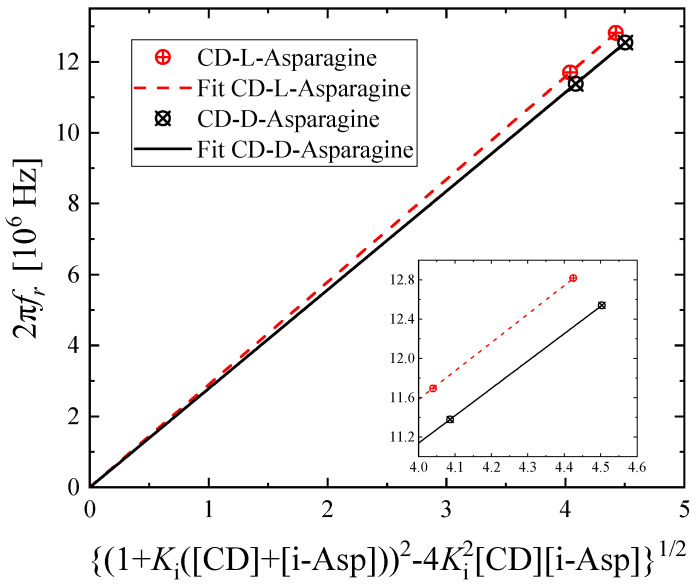
The dependence of the relaxation frequency on the initial concentration of the reactants for both systems. The inset shows a closer examination of the goodness of fit.

From these equations, the reaction constant *K_i_*, and the backward rate constant *k_b,i_* with *i* = d, l can be calculated as:

(6) $$2\pi f_{r,D}=k_{b,D}\{(1+K_{D}(\left[\beta -CD\right]+\left[D-Asp\right]){)}^{2}-4K_{D}^{2}\left[\beta -CD\right]\left[D-Asp\right]{\}}^{\frac{1}{2}}$$

and

(7) $$2\pi f_{r,L}=k_{b,L}\{(1+K_{L}(\left[\beta -CD\right]+\left[L-Asp\right]){)}^{2}-4K_{L}^{2}\left[\beta -CD\right]\left[L-Asp\right]{\}}^{\frac{1}{2}}$$

where *K_i_* is equal to *k_f,i_*/*k_b,i_* with *i* = d, l, and [β-CD], [d-Asp], and [l-Asp] are the initial concentrations. The dependence of the relaxation frequency on the initial concentration of the reactants is presented in Figure 4. The above equations are valid only if the concentration of the acceptor is greater than or equal to that of the receptor [20]. In Figure 4, only the experimental points that fulfilled this requirement were used. The values of *K_i_* and *k_b,i_* with *i* = d, l were adjusted accordingly to adequately fit the experimental data with a linear equation using the least squares method. The goodness of fit procedure is more easily examined on the inset of Figure 4. The obtained values corresponding to the best linear fitting going through an intercept equal to zero are presented in Table 1 for the inclusion complexes of β-cyclodextrin with each one of the chiral forms of asparagine.

Despite both inclusion complexes exhibiting a close resemblance, the β-CD–d-Asp complex appears more stable than the l-asparagine counterpart, while the latter reveals higher rate constants. Furthermore, the fact that the rate constant values are high for both systems implies that both binding and release of the amino acid from the β-cyclodextrins cavity are performed with considerable ease. This is reasonable considering their enhanced mobility due to their molecular size [20].

The standard volume change during the reaction can also be evaluated directly from the acoustic parameters [16]. The maximum absorbance per wavelength (*μ_max_*) is given by:

(8) $$\mu_{max}={0.5Af}_{r}u$$

where *A*, *f_r_*, and *u* are the amplitude of the relaxation, its characteristic frequency, and the sound velocity in the liquid sample, respectively. The volume change during a reaction is associated with the maximum absorbance per wavelength (*μ_max_*) for each complexation process through equation [16]:

(9) $$\mu_{max,D}=\frac{\pi \rho u_{D}^{2}}{2RT}(\frac{1}{{\left[D-Asp\right]}_{eq}}+\frac{1}{{\left[\beta -CD\right]}_{eq}}+\frac{1}{{\left[\beta -CD—D-Asp\right]}_{eq}}{)}^{-1}{\left(\Delta V\right)}_{D}^{2}$$

and

(10) $$\mu_{max,L}=\frac{\pi \rho u_{L}^{2}}{2RT}(\frac{1}{{\left[L-Asp\right]}_{eq}}+\frac{1}{{\left[\beta -CD\right]}_{eq}}+\frac{1}{{\left[\beta -CD—L-Asp\right]}_{eq}}{)}^{-1}{\left(\Delta V\right)}_{L}^{2}$$

where *R* denotes the gas constant and *T* is the absolute temperature. The rest of the symbols have their usual meaning.

**Figure 5:**
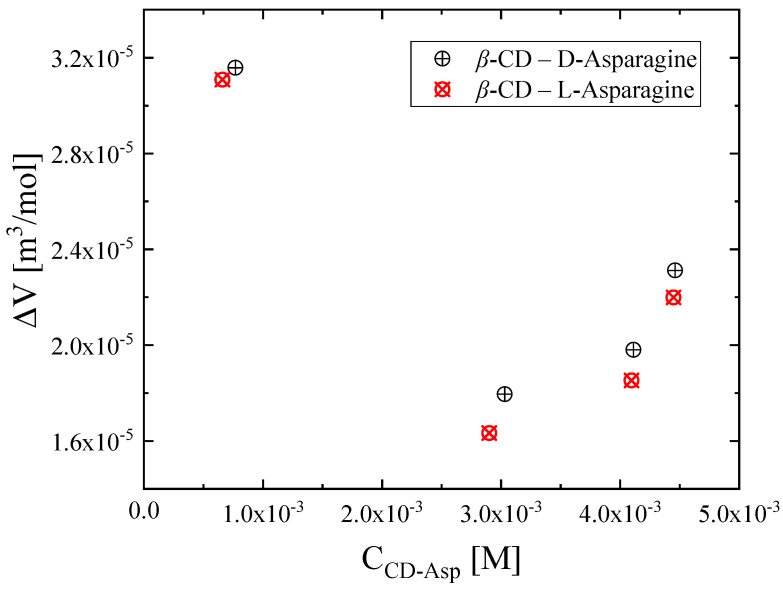
The standard volume change accompanying the reaction as a function of the formed complex concentration.

The results are shown in Figure 5, where it seems that there is a notable change when the concentration of β-cyclodextrin exceeds the concentration of the amino acid. This change is due to the 1:1 stoichiometry of the reaction. Moreover, among the two complexes, the one containing d-asparagine reveals a larger volume change for all concentrations, indicating a more effective encapsulation compared to its counterpart.

Differences in the hydrophobic part in the guest molecules may strongly affect encapsulation. More specifically, the higher the hydrophobicity of the guest molecule, the easier the incorporation into the β-CD cavity is. Nevertheless, the two enantiomers of asparagine reveal almost the same hydrophobic properties, and thus, the standard volume change during the complexation reaction appears comparable [18]. Additionally, a factor that may affect the magnitude of the standard volume change is the nature of the amino acids, e.g., having a branched hydrocarbon chain, which may not allow a deeper encapsulation (penetration) into β-cyclodextrin [18]. In our case, both enantiomers bear similar structure, and the cavity size of β-CD is large enough to distinguish among the two enantiomers of asparagine results in comparable volume change.

**Figure 6:**
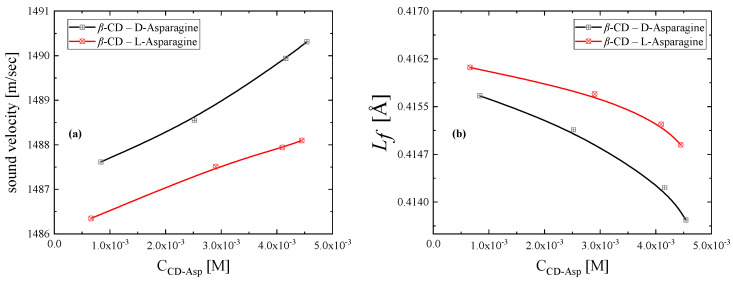
Speed of sound (**a**) and free intermolecular distance (**b**) as a function of the formed complex concentration.

The sound velocity in a fluid is a valuable thermo-physical property that can be used as a diagnostic tool in the study of inclusion complexation. As shown in Figure 6a, the formation of a larger amount of the inclusion complex leads to a monotonous increase in the sound speed for both cases. This behavior is expected on the grounds that the structures created during complexation are more rigid, thus allowing for faster propagation of sound in the medium. The rigidity of the structure affects the dynamic response of the system, and thus, a sound wave will travel faster in a more compact structure. Furthermore, the sound velocity is higher for d-asparagine compared to the l-inclusion complex due to the better encapsulation of d-asparagine, which creates more rigid structural species [21].

Another valuable parameter that can be calculated is the free intermolecular distance *L_f_*, which is given by [22]:

(11) $$L_{f}=K\sqrt{\kappa_{s}}$$

where K is the so-called Jacobson parameter and κ_s_ is the adiabatic compressibility that can be estimated as $\kappa_{s}={\left(\rho u^{2}\right)}^{-1}$. The calculated values of the free intermolecular distance are illustrated in Figure 6b, where a monotonous decreasing trend is observed with increasing complex content in the solution. This result is expected considering that progressively more molecules are encapsulated in the cavity of β-cyclodextrin, reducing the space between molecules, and thus the free intermolecular distance decreases.

### 2.3. Thermodynamic Evaluation of the Complexation Mechanism

**Figure 7:**
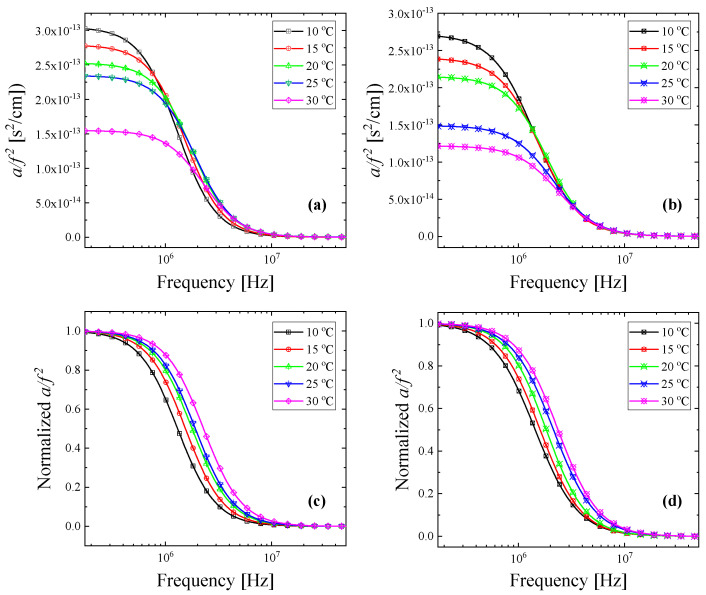
Sound absorption coefficient (*a/f*^2^) as a function of frequency for all temperatures studied for d-asparagine (**a**) and l-asparagine (**b**) solutions with β-cyclodextrin corresponding to concentrations of [β-CD] = 5 mM, [d-Asp] = 5 mM, and [l-Asp] = 5 mM. Normalized *a/f*^2^ values as a function of frequency for d-asparagine (**c**) and l-asparagine (**d**). The normalized representation facilitates the observation of the characteristic frequency (*f_r_*) shift with temperature.

The experimental absorption coefficients as a function of frequency for all temperatures studied for β-cyclodextrin with a d-asparagine solution with concentrations of [β-CD] = 5 mM and [d-Asp] = 5 mM and an l-asparagine solution with concentrations of [β-CD] = 5 mM and [l-Asp] = 5 mM are illustrated in Figure 7a,b, respectively. The relaxation amplitude increases with temperature for both systems. The normalized representation of the acoustic spectra shown in Figure 7c,d facilitates the observation of the characteristic frequency (*f_r_*) blue shift with temperature. Continuous lines correspond to the relaxation curves after the fitting for all temperatures, while symbols represent experimental points. It seems from the goodness of fit that the Debye-type profile efficiently describes the experimental data. From this procedure, we quantitatively acquired the variation in relaxation amplitude and the characteristic frequency for each system with temperature.

By means of Eyring’s equation, the activation enthalpy ΔH* and activation entropy ΔS* can be easily estimated from the temperature dependence of the characteristic relaxation frequency for both complexes [23]:

(12) $$ln\left(\frac{f_{r}}{T}\right)=-\frac{{\Delta H}^{*}}{1000R}\left(\frac{1000}{T}\right)+\left[\frac{{\Delta S}^{*}}{R}+ln\left(\frac{k_{B}}{2\pi h}\right)\right]$$

where *k_B_* and *h* are Boltzmann’s and Planck’s constants, respectively.

**Figure 8:**
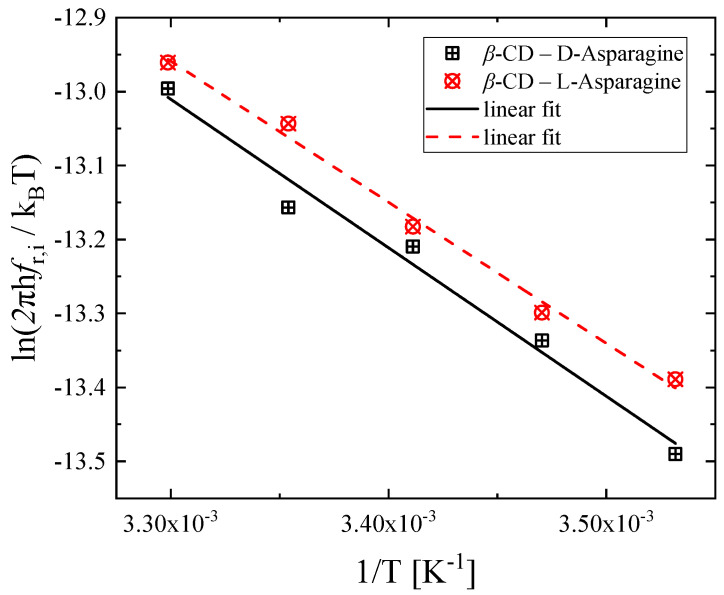
Variation of ln2πhfr,ikΒΤ as a function of 1/T for the β-CD—d-Asp and β-CD—l-Asp inclusion complexes.

The ln2πhfr,ikΒΤ vs. *1*/*T* plot presented in Figure 8 exhibits a clear linear dependence. From this graph, one can determine the activation enthalpy ΔH* and activation entropy ΔS* from the slope and the intercept for both systems that are summarized in Table 2.

The activation enthalpy and entropy presented in Table 2 demonstrate only minor differences between the two systems. Nevertheless, in the d-asparagine inclusion complex, the activation enthalpy is higher, and the activation entropy is lower than that of the l-asparagine complex. The difference between the enthalpies of the two enantiomers would be significantly greater for larger guest molecules with larger dimensions or for inclusion in host molecule with smaller cavities like α-cyclodextrin [24]. In our case, both asparagine enantiomers are flexible small-sized molecules that exhibit small conformational changes, and for this reason, the alteration from the d-Asp to l-Asp configuration does not affect the complexation process.

### 2.4. Vibrational Modes—Short-Range Structure

Vibrational spectroscopy is a valuable technique for examining molecular-level structural changes in short-range order. We recorded the infrared absorption spectra of the host β-cyclodextrin molecule and the guest d-asparagine and l-asparagine molecules in the solid state under ambient pressure and temperature conditions. The complexes of β-CD—d-Asp and β-CD—l-Asp were received in the solid state from the solution by applying the layering technique. Subsequently, their IR spectra was obtained in the same manner. The solid state was chosen for characterization because of its scientific significance and practical appeal. It is particularly suitable for structural analysis, examining molecular packing and crystal behavior, and investigating applications in materials science.

**Figure 9:**
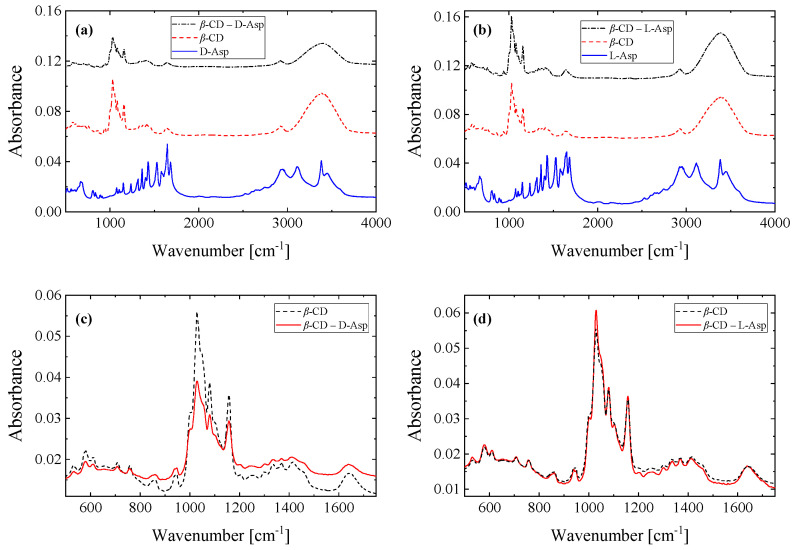
Infrared absorption spectra of (**a**) d-asparagine, β-cyclodextrin, and their complex and (**b**) l-asparagine, β-cyclodextrin, and their complex. The spectra in the fingerprint spectral region of β-cyclodextrin and the inclusion complex with d-asparagine (**c**) and l-asparagine (**d**).

Figure 9a,b shows the spectra of the host, guest, and inclusion complex molecules for both d- and l-enantiomers of asparagine. All spectra were normalized to allow for quantitative comparison. Special attention was paid to the fingerprint spectral region (Figure 9c,d) with the aim of verifying the formation of the d- and l-asparagine inclusion complexes with β-cyclodextrin. For both cases, the spectra of the complexes exhibit a close resemblance with the spectrum of pure β-cyclodextrin compared to the spectrum of pure asparagine enantiomers. The finding that the spectrum of the inclusion complex appears similar to the spectrum of the host to a greater extent than the spectrum of the guest molecule has been reported in the past for analogous systems [25].

Despite the extended spectral similarities, various minor absorbance variations, peak shape modifications, and frequency shifts are observed in the spectra of the complexes relative to pure β-cyclodextrin (Figure 9c,d) due to the encapsulation of the amino acid in β-cyclodextrin.

Starting from the high-frequency region, the infrared spectrum of β-CD is dominated by a characteristic broad band with a maximum located at ~3300–3400 cm^−1^, which is assigned to the stretching modes of hydroxyl group (O–H). The bands at ~2900 cm^−1^ are due to C–H asymmetric stretching modes. The bands near ~1415 cm^−1^, ~1645, and ~1334 cm^−1^ are related to the O–H bending vibrations. The ~1152 cm^−1^ peak is due to the C–O–C glycosidic symmetric stretching vibration. In lower frequencies, the ~1077 and ~1021 cm^−1^ bands are assigned to C–C and C–O stretching vibrations, respectively. Finally, the ~938 cm^−1^ mode is ascribed to the C–H stretching vibrations of the cyclodextrin ring [26,27].

The infrared spectra of asparagine enantiomers are dominated by the spectral fingerprints of the functional group participating in the structure, namely the CH_2_, NH_2_, C=O, and COOH groups [28]. The symmetric and asymmetric stretching modes of CH_2_ are observed in the 2800–3030 region. The scissoring and deformation modes of CH_2_ are shown at 1433 and 1400 cm^−1^, respectively. The symmetric and asymmetric N–H stretching modes of the NH_2_ functional group appear in the region of 3500–3300 cm^−1^. The scissoring mode of NH_2_ is observed between 1650 and 1529 cm^−1^. Finally, the bands at ~1150 and ~1121 cm^−1^ are attributed to rocking modes of NH_2_. The band with medium absorbance at 1673 cm^−1^ is assigned to the C=O stretching mode. The ~1681 cm^−1^ band is assigned to the C=O stretching vibration of the COOH functional group. Furthermore, the hydroxyl group of COOH that is non-hydrogen bonded exhibits vibrations with frequencies in the range of the 3700–3600 cm^−1^ spectral region. The vibrations of the hydrogen bonded hydroxyl groups of COOH are observed at lower frequencies, in the 3550–3200 cm^−1^ range, and are broader and more intense. The corresponding in-plane and out-of-plane O–H bending vibrations appear at ~1426 and ~915 cm^−1^, respectively [28].

All the intense and sharp bands attributed to β-CD were detected in the spectra of the inclusion complexes. The strong peaks of asparagine enantiomers observed in the 1000–2000 cm^−1^ frequency range almost disappeared in the spectra of complexes. By this outcome, we infer that both asparagine enantiomers were contained within the cavity of β-CD through van der Waals forces and hydrophobic interactions. As a result of the asparagine enantiomer incorporation in β-cyclodextrin, all the vibrational modes of the latter were shifted to higher or lower frequencies, confirming the formation of the inclusion complex and that the complex is not a physical mixture.

**Figure 10:**
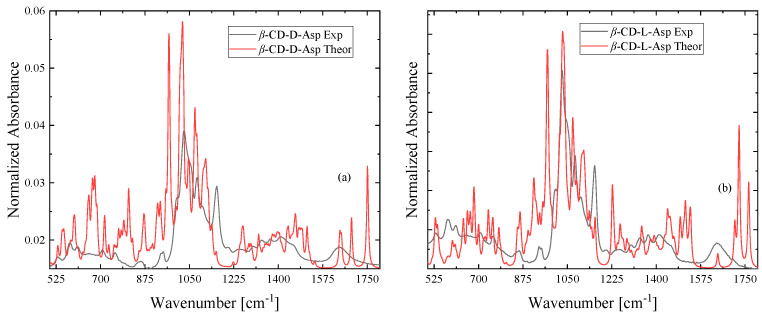
Theoretical and experimental IR spectra of the d-asparagine (**a**) and l-asparagine (**b**) inclusion complexes with β-cyclodextrin.

Figure 10a,b presents the experimental and theoretically calculated infrared spectra of the d-asparagine and l-asparagine inclusion complexes for comparison. It seems that the spectra are isomorphous for both cases. Numerous bands coincide in frequency and vary in their relative intensities at low frequencies. Even though the theoretical calculation was performed in a vacuum without considering any intermolecular interaction, the comparison further supports the successful encapsulation of the two forms of asparagine in the cavity of β-cyclodextrin. These findings emphasize the effectiveness of FT-IR analysis, enhanced by DFT calculations, in uncovering the molecular behavior and interaction dynamics of β-CD complexes. Furthermore, these investigations produced consistent findings, revealing notable spectral changes in the complex, such as frequency shifts, intensity variations, and modifications in peak shapes compared to β-CD and the guest molecule. These changes highlight the specific interactions and structural adjustments occurring within the complex.

## 3. Materials and Methods

### 3.1. Solutions and Density Measurements

β-Cyclodextrin (purity: 99%, Sigma-Aldrich, Burlington, MA, USA) and the two d- and l-enantiomers of asparagine (purity: 99%, Fluka, Charlotte, NC, USA) were dissolved in triply distilled water to prepare the final solutions without any further purification.

The corresponding molar concentrations of the prepared solutions are presented in Table 3. The concentration of the amino acids in the solutions remained constant at 5.0 × 10^−3^ M, while the concentration of cyclodextrin varied from 1.0 × 10^−3^ to 15.0 × 10^−3^ M. The density of the solution was measured by means of a temperature-controlled pycnometer (DMA 40, Anton Paar, Ostfildern, Germany) with an accuracy of ±0.0001 g/cm^3^. The inclusion complex of both asparagine enantiomers was received in the solid state by applying the so-called layering technique. More technical details concerning this methodology have been reported in [15].

### 3.2. Ultrasonic Relaxation Spectroscopy

The absorption coefficient of the ultrasound was estimated by using the parallel-path pulse technique. For each measurement, less than 2 mL of the liquid sample was placed in a temperature-controlled acoustic cell with cylindrical geometry. The cell was positioned between two parallel broadband transducers (V111, Olympus-Evident, Tokyo, Japan) with a central frequency of 10 MHz. To ensure optimal transmission of the ultrasonic wave, a commercial medical couplant was placed between acoustic cell faces and transducers. A pulse generator (TGP3151, TTi, Maisach, Germany) was utilized to trigger one of the piezoelectric elements, which transmitted an acoustic wave at the desired frequency. The ultrasonic signal travelled through the liquid sample and was finally received by the second piezoelectric element serving as a signal receiver. The path length inside the acoustic cell was fixed at 1 cm. The receiving signal was then sent to a digital oscilloscope (TBS 1202B, Tektronix, Beaverton, OR, USA) for monitoring and signal analysis [29]. Possible losses due to the diffraction effect were considered in our measurements and corrected. From the fixed path length of the acoustic cell and the time required for the ultrasonic wave to travel through the cell, we were able to evaluate the sound velocity. This time corresponds to the time interval between two consecutive echoes. The accuracies of the attenuation coefficient and sound velocity were ±5% and ±0.01%, respectively [30]. More about the technical details and experimental protocols of the acoustic measurements can be found elsewhere [31].

### 3.3. Vibrational Spectroscopy

The infrared spectra of pure β-cyclodextrin, d-asparagine, l-asparagine, and the inclusion complexes were recorded in the 400 to 4000 cm^−1^ spectral region by mixing the appropriate amount of the solid with anhydrous KBr in the solid state. The spectrometer used for the IR measurements (FT/IR-4700, Jasco, Tokyo, Japan) was equipped with sealed optics and a DLATGS detector cooled with Peltier elements. For each measurement, a homogenous pellet was prepared by mixing 2 mg of the compound with 100 mg of KBr. The spectral resolution of all measurements was fixed at 2 cm^−1^. The spectrum of each sample corresponded to the average of 24 individual scans. The spectrum of the pure KBr pellet was recorded as background as in [15].

### 3.4. DFT and Molecular Docking Calculations

The molecular structure of β-cyclodextrin was fetched from the rcsb protein data bank (PRD_900012). The structures of d-asparagine (PubChem CID: 439600) and l-asparagine (PubChem CID: 6267) were fetched from PubChem database in digital form. These structures were then optimized by means of the B3LYP functional in conjunction with the 6-311G(d,p) basis set in the framework of the density functional theory (DFT) in a vacuum [32]. The B3LYP functional was selected due to its robust performance and computational efficiency. The polarization function was also employed on all atoms to ensure a detailed description of electron distribution and molecular interactions. Furthermore, the theoretical IR spectra and the molecular volume of all species were also calculated. Regarding the vibrational frequencies, the scaling factor used was 0.967 (accessed on 1 November 2024 from https://cccbdb.nist.gov/vibscalejustx.asp). No imaginary vibrational frequencies were found, indicating that the optimized structures correspond to stable energy minima. This methodology provided a comprehensive understanding of the molecular properties and interactions within the inclusion complexes. The Gaussian 09 W Revision D.01 package was employed for all quantum mechanical calculations.

The formation of the inclusion complexes was theoretically investigated through molecular docking calculations. These calculations were performed by means of the AutoDock 4.2 software [33]. The process began with the initial optimization of β-cyclodextrin and the asparagine enantiomers to ensure their energetic and geometric stability prior to docking. For the docking simulation, firstly, the boundaries of the simulation box were established at 25 Å for each of the three main axes, with grid spacing of 0.375 Å. The optimized structure of β-cyclodextrin was considered as the immobile receptor, while each of the enantiomers of asparagine acting as a ligand was set free to move around the receptor with the maximum number of rotatable bonds permitted within the simulation box. The derived poses were generated with the Lamarckian genetic algorithm (LGA) and the docking score was used to define the best pose in terms of stability [34].

## 4. Conclusions

In the present paper, we have presented a detailed experimental and theoretical study of the complexation of asparagine enantiomers (d-asparagine and l-asparagine) with β-cyclodextrin. The formation of the β-CD—d-Asp and β-CD—l-Asp inclusion complexes was established by means of molecular docking computational investigation. The dispersion of the ultrasonic absorption curves with concentration revealed the presence of a single relaxation process assigned to the inclusion complex formation. The reaction constant and the forward and backward rate constants for the complexation of β-cyclodextrin with the d- and l-forms of asparagine were also determined. The higher equilibrium constant of d-asparagine was reflected in the slightly higher absolute value of the binding energy found by the molecular docking calculations. The standard volume change accompanying the reaction, the sound velocity, and the free intermolecular distance as a function of the complex concentration revealed that among the two complexes, the one containing d-asparagine exhibited a more effective encapsulation in β-cyclodextrin. From the temperature dependence of the acoustic spectra, the activation enthalpy and entropy for the complexation were estimated. For the d-asparagine inclusion complex, the activation enthalpy was higher and the activation entropy lower than that of the l-asparagine complex. The infrared absorption spectroscopy was utilized to verify the proposed structural mechanism. The comparison among the spectra of host, guest enantiomers, and inclusion complexes exposed various minor absorbance variations, peak shape modifications, and frequency shifts due to the encapsulation of the amino acid in β-cyclodextrin. Furthermore, the corresponding theoretical spectra of host, guest enantiomers, and inclusion complexes were determined by means of DFT methodologies. The strong similarity between the experimental and theoretically predicted IR spectra supports the formation of the inclusion complexes.

## Figures and Tables

**Table 1:** The reaction constant and the forward and backward rate constants for the complexation of β-cyclodextrin with the d- and l-forms of asparagine.

	*Κ* [Μ^−1^]	*k_f_* [mol^−1^Ls^−1^]	*k_b_* [s^−1^]
β-CD—d-Asp	785	2.19 × 10^10^	2.78 × 10^7^
β-CD—l-Asp	766	2.21 × 10^10^	2.89 × 10^7^

**Table 2:** The activation enthalpy and entropy for the β-cyclodextrin complexation with asparagine enantiomers.

	ΔH* (kcal/mol)	ΔS* (cal/mol·K)
β-CD—d-Asp	3.99 ± 0.31	−12.69 ± 1.09
β-CD—l-Asp	3.79 ± 0.18	−13.24 ± 2.62

**Table 3:** Molar concentrations of the prepared solutions.

	β-CD [mM]	d-Asparagine [mM]	l-Asparagine [mM]
Sample 1	1.0	5.0	-
Sample 2	5.0	5.0	-
Sample 3	10.0	5.0	-
Sample 4	15.0	5.0	-
Sample 5	1.0	-	5.0
Sample 6	5.0	-	5.0
Sample 7	10.0	-	5.0
Sample 8	15.0	-	5.0

## Data Availability

Data will be made available on request.
